# Protective effects of menthol against sepsis-induced hepatic injury: Role of mediators of hepatic inflammation, apoptosis, and regeneration

**DOI:** 10.3389/fphar.2022.952337

**Published:** 2022-08-30

**Authors:** Asmaa I. Matouk, Mahmoud El-Daly, Heba A. Habib, Shaymaa Senousy, Sara Mohamed Naguib Abdel Hafez, AlShaimaa W. Kasem, Waleed Hassan Almalki, Abdulaziz Alzahrani, Ahmed Alshehri, Al-Shaimaa F. Ahmed

**Affiliations:** ^1^ Department of Pharmacology and Toxicology, Faculty of Pharmacy, Minia University, Minya, Egypt; ^2^ Department of Pathology, Faculty of Medicine, Minia University, Minya, Egypt; ^3^ Department of Histology and Cell Biology, Faculty of Medicine, Minia University, Minya, Egypt; ^4^ Department of Pharmacology and Toxicology, Umm Al-Qura University, Makkah, Saudi Arabia; ^5^ Department of Pharmacology and Toxicology, College of Clinical Pharmacy, ‬AlBaha University, Al Bahah, Saudi Arabia; ^6^ Department of Pharmacology and Toxicology, Faculty of Pharmacy, Northern Border University, Rafha, Saudi Arabia

**Keywords:** clp, hepatoprotection, tNF-alpha, PCNA, apoptosis

## Abstract

Liver dysfunction in sepsis is a major complication that amplifies multiple organ failure and increases the risk of death. Inflammation and oxidative stress are the main mediators in the pathophysiology of sepsis. Therefore, we investigated the role of menthol, a natural antioxidant, against sepsis-induced liver injury in female Wistar rats. Sepsis was induced by cecal ligation and puncture (CLP). Menthol (100 mg/kg) was given intragastric 2 h after CLP. Blood samples and liver tissues were collected 24 h after surgery. Menthol significantly (*p* < 0.05) attenuated the sepsis-induced elevation in serum liver enzymes and improved the hepatic histopathological changes. Menthol treatment significantly (*p* < 0.05) decreased hepatic levels of tumor necrosis factor-alpha, malondialdehyde, total nitrite, and cleaved caspase-3. It restored the hepatic levels of superoxide dismutase and reduced glutathione. Additionally, menthol significantly (*p* < 0.05) increased hepatic levels of B-cell lymphoma 2 (Bcl-2); an anti-apoptotic factor, and proliferating cell nuclear antigen (PCNA), a biomarker of regeneration and survival. Our results showed the therapeutic potential of menthol against liver injury induced by sepsis.

## 1 Introduction

Sepsis is a global health issue and a leading cause of death, especially in patients with low immunity, including children, the elderly, immunocompromised individuals, and intensive care unit patients (Fleischmann et al., 2016; Rudd et al., 2017; Dou et al., 2019a). Sepsis is a condition of life-threatening organ dysfunction mainly due to the host’s immune response to infections (Seymour et al., 2016). Sepsis induces damage to many organs, including the lungs, heart, and liver. However, the liver is more vulnerable and often affected earlier by sepsis (Saini et al., 2022). Hepatic dysfunction during sepsis incidence ranges from 34% to 46% and is considered a powerful predictor of sepsis-related mortality. The risk of death due to sepsis-induced hepatic failure is 54%–68% (Strnad et al., 2017; Woznica et al., 2018), which is higher than the death rate due to sepsis-induced lung dysfunction (Yan and Li, 2014). The liver is a key component of the primary defense line against infection. Early in sepsis, Kupffer cells play an essential role in the removal of bacteria and endotoxins through the release of proinflammatory cytokines such as interleukin (IL)-1β, IL-6, IL-18, and tumor necrosis factor-alpha (TNF-α) (Traeger et al., 2010). Although these cytokines help eradicate the pathogens, they also provoke liver damage and stimulate the release of other inflammatory mediators in a process known as a cytokine storm. This eventually leads to a systemic inflammatory response and multiple organ damage (Lelubre and Vincent, 2018; Woznica et al., 2018). Sepsis leads to a profound reduction in hepatic functions, including the dysregulation of carbohydrate, lipid, and protein metabolism, decreased synthesis and release of bile and coagulation factors, impaired defense against pathogens, increased production of inflammatory mediators, and the amplification of other organs’ failure (Srivastava and Gimson, 2013; Yan and Li, 2014; Strnad et al., 2017). Understanding the underlying mechanisms of hepatic dysfunction in sepsis is still a challenge. The pathogenesis of sepsis-induced liver injury involves many factors, including bacterial toxins, septic shock induced-hypotension, hepatic hypoperfusion (Spapen, 2008; Henrion, 2012), impairment of the endothelial function of hepatic microvasculature (La Mura et al., 2013), production of reactive oxygen species (ROS), and proinflammatory cytokines (Strnad et al., 2017). Increased ROS production and inflammation remain the hallmarks of hepatic damage in sepsis. Thus, efforts to control sepsis-induced liver injury focused on drugs with antioxidant and anti-inflammatory effects to prevent multiple organ damage and decrease mortality (Ahmed et al., 2020; Al-Kadi et al., 2020).

Menthol, the main component of *Mentha arvensis L. (Lamiaceae) and Mentha piperita L.* essential oil, is a naturally occurring monoterpene widely employed in different pharmaceutical formulations as a flavoring agent and an oral care product. Menthol has multiple biological activities, including antinociceptive (Pergolizzi et al., 2018), antispasmodic (Amato et al., 2014), local anesthetic (Galeotti et al., 2001), antibacterial, and antifungal effects (Sabzghabaee et al., 2011). Accumulating evidence showed that menthol modulates the production of TNF-α and interleukins, decreases ROS generation, and enhances the antioxidant enzyme activity in different animal models (Janbaz and Gilani, 2002; Rozza et al., 2014; Bastaki et al., 2018; Rozza et al., 2021). The anti-inflammatory and antioxidant effects of menthol proved protective against paracetamol- and carbon tetrachloride (CCl_4_)-induced hepatic toxicity (Janbaz and Gilani, 2002), acetic acid-induced colitis (Bastaki et al., 2018), gastric ulceration (Rozza et al., 2013; Rozza et al., 2014), Parkinson’s disease (Du et al., 2020), skin wounds (Rozza et al., 2021), and Freund adjuvant-induced peripheral inflammation (Hilfiger et al., 2021). Menthol, a natural product with minimal side effects, is available at low costs and possesses hepatoprotective effects. However, whether or not menthol can alleviate sepsis-induced liver injury has not yet been studied. Hence, this study aimed to explore the therapeutic potential and possible protective mechanisms of menthol against sepsis-induced hepatic dysfunction.

## 2 Materials and methods

### 2.1 Animals and drugs

Female Wistar rats (8–10 weeks old) weighing (200–220 g) were obtained from El-Nahda University Animal House (Beni-Suef, Egypt) for this study. Rats were housed under specific pathogen-free conditions on a 12-hrs light-dark cycle with free access to regular rat chow (El-Nasr Company, Abou Zaabal, Cairo, Egypt) and tap water. Rats were left for 1 week as an acclimatization period prior to the experiment. All experimental procedures were approved by The Commission on the Ethics of Scientific Research, Faculty of Pharmacy, Minia University, Egypt (ES02/2020). Menthol was purchased from (Sigma-Aldrich Inc., United States).

### 2.2 Induction of sepsis

Cecal ligation and puncture (CLP), a precise and commonly used sepsis model, was used to induce sepsis as previously prescribed (Nemzek et al., 2008; Ahmed et al., 2020). Rats were anesthetized with ketamine (50 mg/kg) and xylazine (10 mg/kg). A longitudinal abdominal incision was made in the lower left quadrant of the body to expose the cecum. 0.3-mm silk surgical suture thread was used to ligate the cecum just below the ileo-cecal valve, and the ligated part was punctured twice with an 18-gauge needle to ensure the same degree of severity of sepsis in all groups. The percentage of the ligated portion (75%) was kept constant in all groups. Finally, the cecum was gently returned to its place in the abdominal cavity, and the incision was sutured. All animals received normal saline [3 ml/100 g, subcutaneous (S.C.)] to help for resuscitation after surgery. After the surgical procedure, the rats exhibited symptoms of illness, including piloerection, diarrhea, and malaise according to Morton and Griffiths (Morton and Griffiths, 1985). In addition, similar to our previous studies (Ibrahim et al., 2020a; Al-Kadi et al., 2020; Al‐Kadi et al., 2021), ischemia and inflammation in the ligated cecum confirmed the induction of sepsis in CLP rats (Supplementary Figure S1).

### 2.3 Survival study

Female Wistar rats (200–220 g) were randomly assigned to four groups (*n* = 10, each): sham group, the rats were exposed to all the surgical procedures for the induction of sepsis, except for the ligation and puncture steps. They were given vehicle (1 ml water/kg, I. G) 2 hours after surgery; sham-menthol group, the rats of this group are sham rats that received intragastric (I.G.) menthol (100 mg/kg) dissolved in water (L ml/kg); sepsis group, the rats of this group were exposed to CLP then received the vehicle (1 ml water/kg, I. G) 2 h after CLP surgery; and sepsis-menthol group, the rats of this group were exposed to CLP then received menthol (100 mg/kg, I. G) 2 h after the surgery. All rats were monitored for 7 days to assess the mortality rate. The sample size for the survival analysis was determined according to previous studies in our lab (Ibrahim et al., 2020a; Al‐Kadi et al., 2021) and by others (Chen et al., 2021).

### 2.4 Experimental groups

Thirty female rats were randomly divided into four groups: Group 1 (sham group, *n* = 6), Group 2 (sham-menthol, *n* = 6), Group 3 (sepsis group, *n* = 12), and Group 4 (sepsis-menthol, *n* = 6). All animals received either vehicle (Group 1 and 3) or menthol (100 mg/kg, I. G; Group 2 and 4) 2 h after surgery to ensure the complete recovery from anesthesia and allow I.G. administration. The time of the drug intervention was chosen based on our preliminary study and previous reports (Aksoy et al., 2014; Ibrahim et al., 2020a; Al-Kadi et al., 2020; Al‐Kadi et al., 2021). Our preliminary studies showed the best survival results in the CLP septic rats that received 100 mg/kg menthol (Supplementary Figure S2). Furthermore, this dose was in the range previously reported to have anti-inflammatory, antioxidant, and anti-apoptotic effects in other models (Janbaz and Gilani, 2002; Ghasemi-Pirbaluti et al., 2017; Hilfiger et al., 2021).

Rats of all groups were sacrificed 24 h after surgery and blood samples were then collected by cardiac puncture. A segment of the medial lobe from each animal’s liver was fixed in 10% buffered formalin solution for 24 h, and prepared for histopathological and immunohistochemical examination. Other liver samples were flash-frozen in liquid nitrogen and stored at -80°C for further assessments.

### 2.5 Assessment of hepatic function

For early detection of hepatic dysfunction, serum levels of Glutamic–Pyruvic Transaminase (GPT; ALT) (EC2.6.1.2) and Glutamic–Oxaloacetic Transaminase (GOT; AST) (EC2.6.1.1) were determined by colorimetric assay kits (Diamond Diagnostics, Cairo, Egypt) based on the method of Reitman and Frankel as per the manufacturer’s instructions (Reitman and Frankel, 1957). The measuring range of ALT and AST were up to 94 and 89 U/L, respectively. For quantitation of AST, the samples were diluted ten times, then the results were multiplied by 10.

### 2.6 Assessment of hepatic histopathological changes

Specimens from formalin-fixed liver tissues were processed for routine paraffin embedding. Sections (5-μm-thickness) were stained with haematoxylin and eosin (H&E). A CCD digital camera adapted to BX51 microscope (Olympus, Japan) was used to capture images at ×400 magnification. ImageJ software was used for semi-quantitative analysis. The parameters used to assess histopathological changes included dilated central veins, sinusoidal congestion, hepatocyte necrosis, and hepatocyte fatty changes (Jensen, 2008). All histopathological assessments were done by a histopathologist blind to the treatment.

### 2.7 Assessment of hepatic oxidative stress and antioxidant enzyme activity

To assess the liver’s oxidative stress, levels of malondialdehyde (MDA), a product of lipid peroxidation and an index of oxidative stress, were calorimetrically measured based on the Buege method (Buege and Aust, 1978). Levels of total nitrite were determined calorimetrically based on the Griess assay method previously prescribed (Moorcroft et al., 2001). To assess the antioxidant enzyme activity, hepatic reduced glutathione (GSH) levels were determined by colorimetric measurement of 5-thio-2-nitrobenzoic acid, which is produced after the reduction of Ellman’s reagent (5,5-dithio-bis-2-nitrobenzoic acid) by the sulfahydryl (–SH) group of GSH (Beutler et al., 1963). Hepatic superoxide dismutase (SOD) levels were determined according to a previously described method (Marklund and Marklund, 1974) by measuring the amount of SOD enzyme that inhibits the autoxidation of pyrogallol by 50%.

### 2.8 Immunohistochemical determination of hepatic tumor necrosis factor-alpha, cleaved caspase-3, B-cell lymphoma 2, and proliferating cell nuclear antigen

Liver sections (5-μm-thick) were obtained from representative formalin-fixed, paraffin-embedded blocks and transferred to adhesive slides. After the deparaffinization and dehydration of the sections, endogenous peroxidase activity was blocked by incubation with hydrogen peroxide. Microwave treatment in sodium citrate buffer, pH 6, was used for antigen retrieval. Tissue sections were then incubated with rabbit anti-cleaved caspase-3 (catalog number A19664, ABclonal, MA, United States), mouse anti-proliferating cell nuclear antigen (PCNA) (catalog number A9909, ABclonal, MA, United States), rabbit anti-B-cell lymphoma 2 (Bcl-2) (catalog number A19693, ABclonal, MA, United States), or rabbit anti-TNF-α (catalog number A11534, ABclonal, MA, United States). A negative control experiment was done using the same steps but without the addition of the primary antibody to ensure the reaction specificity. Sections were then incubated with a biotinylated secondary antibody for 30 min at room temperature. An avidin-biotin complex immunoperoxidase system was used to visualize the reaction using 3,3′-diaminobenzidine (DAB) as a chromogen. Sections were counterstained using hematoxylin, then dehydrated, cleared, and mounted with distyrene, plasticizer, and xylene (DPX). The mean surface area fractions of anti-cleaved caspase-3, PCNA, Bcl-2, and TNF-α immuno-positive cells were measured using ImageJ software (version 1.51 k, Wayne Rasband, National Institutes of Health, United States) by a pathologist blind to the experimental groups.

### 2.9 Statistical analysis of data

All statistical analyses were performed using GraphPad Prism (version 7.0; San Diego, CA, United States). Values were expressed as mean ± S.E.M. Shapiro-Wilk normality test was used to test the normality of the data. All data followed a normal distribution. One-way analysis of variance (ANOVA) test was used to test the significance of the results. Tukey’s post hoc test was used for multiple comparisons. Survival analysis was performed using the Log-rank (Mantel-Cox) test. The results were considered statistically significant if the probability (*p*)-values were <0.05. Correlation analysis was carried out by calculating the Pearson correlation coefficient (r). If r <|0.3|, the correlation is considered weak. If r is between |0.3| and |0.7|, this indicates a moderate correlation. If r >|0.7|, this indicates a strong correlation.

## 3 Results

### 3.1 Menthol improved survival in septic rats

**FIGURE 1:**
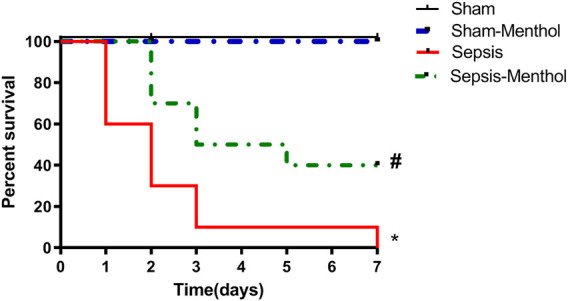
Effect of menthol on CLP-induced mortality. Induction of sepsis by CLP model resulted in 0% survival at the end of the 7^th^ day. Treatment with menthol (100 mg/kg, I. G) 2 h after CLP improved the survival by 40% at the end of the 7^th^ day. The sham group and sham-menthol (100 mg/kg, I. G) group had no mortality throughout the study. Data are presented as a percentage of survival of rats (*n* = 10 per group). * significant difference from the sham group at *p* ˂0.05. # significant difference from the sepsis group at *p* ˂0.05.

Sepsis induction by CLP resulted in 40%, 70%, and 90% mortality by the end of the first, second-, and third-day post-surgery, respectively. On the other hand, administration of menthol (100 mg/kg, I. G) 2 h post CLP resulted in the survival of 100% of rats after the first day, 70% after the second day, and 50% after the third day. All rats in the sham and sham-menthol groups survived the entire seven-day study period. Survival analysis illustrated a significant difference (*p* < 0.05) between the sham group and the sepsis group, as well as between the menthol-treated septic rats and the untreated septic rats (Figure 1).

### 3.2 Menthol attenuated sepsis-induced hepatic injury

**FIGURE 2:**
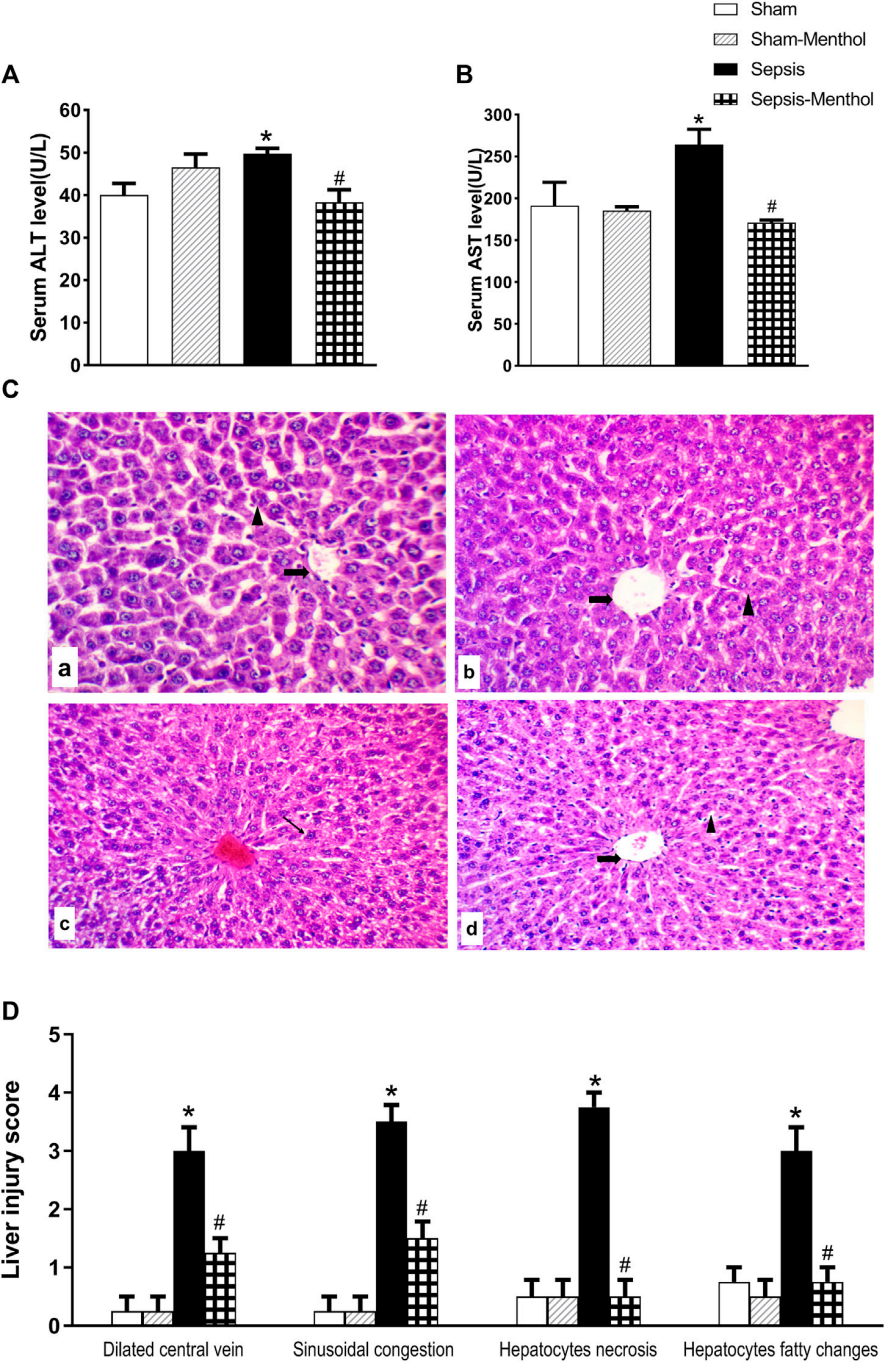
The effect of menthol (100 mg/kg, I.G) on serum levels of ALT (Figure 2A) and AST (Figure 2B) in CLP septic rats. Figure 2C: Photomicrographs showing liver sections from all groups (*n* = 6, each) examined by H&E-stained (200x). The sham groups [sham and sham-menthol; **(A)** and **(B)**] showed normal liver cells (arrow head) with normal central vein (arrow). The liver tissue from the sepsis group **(C)** showed disrupted hepatic cells architecture (arrow head) with dilated central veins (arrow). Liver tissues from the menthol-treated septic rats **(D)** showed normal liver cells. Figure 2D: Scoring the histopathological changes; 0: absent, 1: <25%, 2: >25% and <50%, 3: >50% and <75% and 4: >75% of the entire section showed histopathological alterations. Data represented as a mean score of each group for each observed histopathological alteration. * significant difference from the sham group at *p* ˂0.05. # significant difference from the sepsis group at *p* ˂0.05.

The serum levels of the cytoplasmic liver enzymes, ALT (Figure 2A) and AST (Figure 2B), were significantly (*p* ˂0.05) elevated in the untreated sepsis group when compared with sham group. Treating CLP rats with menthol significantly (*p* ˂0.05) attenuated the sepsis-induced elevation in serum AST and ALT levels. As shown in Figure 2, tissue sections from the sham group and the sham-menthol group exhibited normal histology. However, the sepsis group showed a dilated and congested central vein, surrounded by polygonal-shaped hepatocytes which were separated by congested sinusoidal spaces. Furthermore, perivascular focal necrosis, apoptotic bodies, and moderate fatty changes of peripheral hepatocytes were observed (Figure 2C). The sepsis-menthol group showed a mildly dilated and congested central vein, as well as mild fatty changes in the peripheral hepatocytes. Less inflammation and apoptosis were observed in menthol-treated septic rats. Scores of dilation and congestion of the central vein and sinusoids, as well as necrosis and fatty changes of hepatocytes were significantly (*p* ˂0.05) higher compared to the sham groups. Thus, indicating sepsis-induced deterioration of liver tissues. These changes were significantly (*p* ˂0.05) mitigated by menthol (Figure 2D).

### 3.3 Menthol decreased hepatic oxidative stress in septic rats

Compared with sham-operated rats, liver samples obtained from septic rats showed a significant (*p < 0.05*) elevation in the level of MDA, a biomarker of oxidative stress and lipid peroxidation, as well as the total nitrites (Table 1). The untreated sepsis group showed a significant (*p* < 0.05) reduction in the hepatic antioxidant defense markers, SOD and GSH (Table 1), when compared with the sham-operated rats. Treatment with menthol after CLP surgery significantly (*p* < 0.05) decreased MDA and total nitrite levels. Furthermore, menthol significantly (*p* < 0.05) attenuated the sepsis-induced reduction in hepatic SOD and GSH levels (Table 1).

**TABLE 1:** The effect of menthol (100 mg/kg, I.G.) treatment on hepatic oxidative stress and antioxidant enzyme levels in CLP sepsis model.

Groups	Amount of MDA (nmol/g tissue)	Amount of total nitrite (nmol/g tissue)	SOD activity (U/mg tissue)	GSH (nmol/g tissue)
Sham	0.29 ± 0.09	1.92 ± 0.44	1.64 ± 0.02	10.45 ± 1.03
Sham-Menthol	0.32 ± 0.08	1.87 ± 0.17	2.01 ± 0.70	10.98 ± 1.12
Sepsis	0.75 ± 0.12*	3.22 ± 0.92 *	0.25 ± 0.02*	4.49 ± 1.28*
Sepsis-Menthol	0.49 ± 0.15#	1.71 ± 0.41#	1.33 ± 0.22#	12.18 ± 1.05#

Data was analyzed by ANOVA test, followed by Tukey-Kramer for multiple comparison. Data represent the mean ± SEM of 6 observations; ∗ significant difference from the sham group at *p* ˂0.05. # significant difference from the sepsis group at *p* ˂0.05.

### 3.4 Menthol attenuated sepsis-induced hepatic inflammation and apoptosis

**FIGURE 3:**
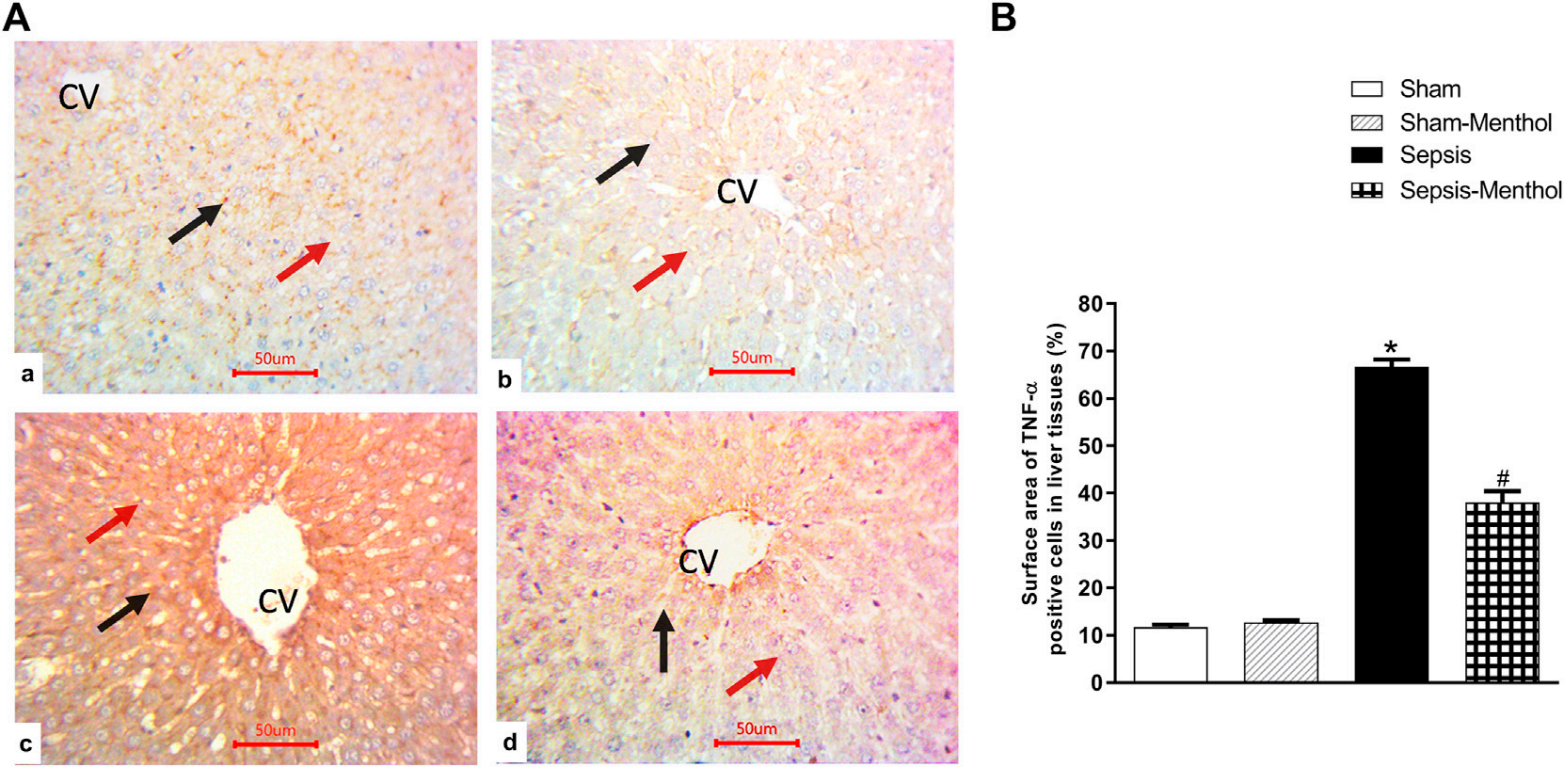
The effect of menthol (100 mg/kg, I.G) on hepatic TNF-α in CLP sepsis model; **(A)**; Representative photomicrographs showing TNF-α immunoreactivity in liver tissue, **(B)**; Bar charts showing semi-quantitative analysis of data in A from sections of the sham, sham-menthol, sepsis, and sepsis-menthol groups. * significant difference from the sham group at *p* ˂0.05. # significant difference from the sepsis group at *p* ˂0.05. Data represented as mean ± S.E (*n* = 6).

**FIGURE 4:**
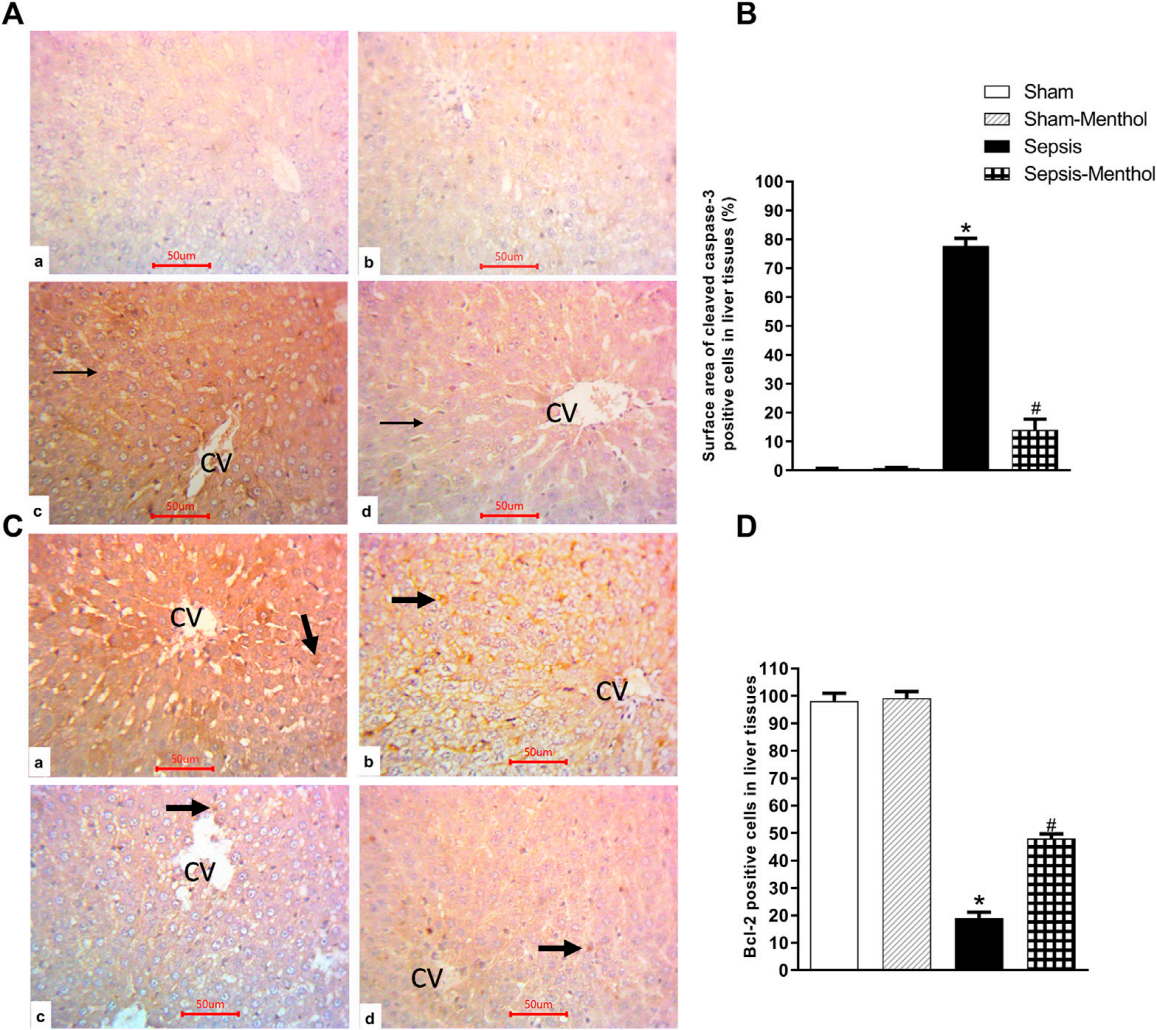
The effect of menthol (100 mg/kg, I.G) on hepatic cleaved caspase-3 and Bcl-2 levels in CLP sepsis model. **(A)**; Representative photomicrographs showing cleaved caspase-3 immunoreactivity in liver tissues. **(B)**; Bar charts showing semi-quantitative analysis of data in A from tissue sections of the sham, sham-menthol, sepsis, and sepsis-menthol groups. **(C)** Representative photomicrographs showing Bcl-2 immunoreactivity in liver tissues. **(D)** Bar charts showing semi-quantitative analysis of data in C from tissue sections of the sham, sham-menthol, sepsis, and sepsis-menthol groups * significant difference from the sham group at *p* ˂0.05. # significant difference from the sepsis group at *p* ˂0.05. Data represented as mean ± S.E (*n* = 6).

Immunohistochemical data revealed a significant (*p* < 0.05) increase in the hepatic expression of the inflammatory mediator TNF-α in the untreated sepsis group (Figure 3). Additionally, untreated septic rats exhibited a significant (*p* < 0.05) upregulation of the apoptotic factor cleaved caspase-3, accompanied by a significant (*p* < 0.05) reduction in the expression of the anti-apoptotic factor Bcl-2 (Figure 4). In contrast, menthol treatment significantly (*p* < 0.05) abrogated the sepsis-induced elevation in hepatic TNF-α levels (Figure 3). Compared to the untreated sepsis group, menthol exhibited remarkable anti-apoptotic activity reflected by a significant (*p* < 0.05) decline in hepatic cleaved caspase-3 expression (Figure 4B) and enhanced expression of Bcl-2 in treated septic rats (Figure 4D).

### 3.5 Menthol promoted hepatocellular regeneration in septic rats

**FIGURE 5:**
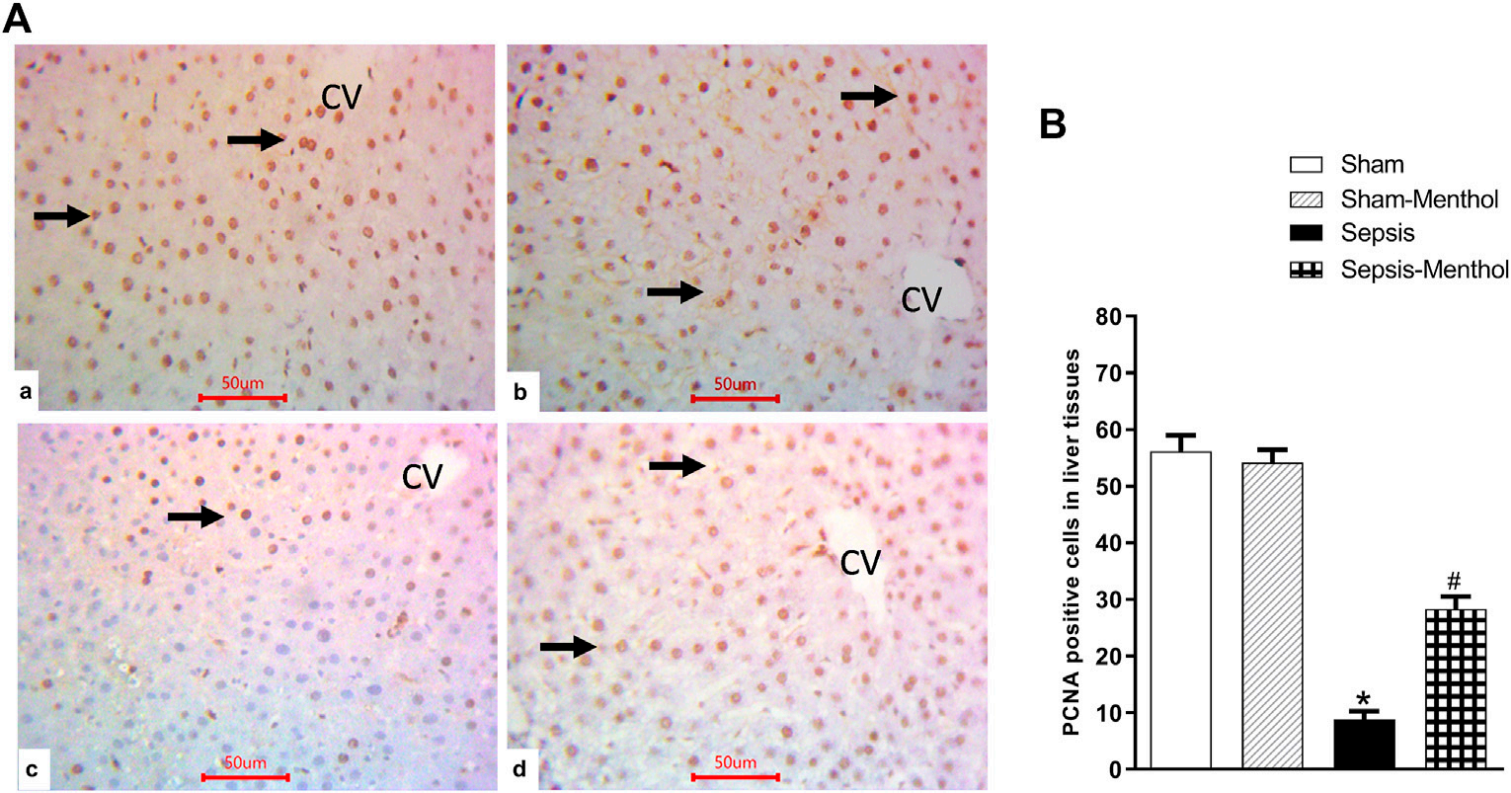
The effect of menthol (100 mg/kg, I.G) on hepatic PCNA levels in CLP sepsis model **(A)**; Representative photomicrographs showing PCNA immunoreactivity in liver tissues. **(B)**; Bar charts showing semi-quantitative analysis of the data from all groups. * significant difference from the sham group at *p* ˂0.05. # significant difference from the sepsis group at *p* ˂0.05. Data represented as mean ± S.E (*n* = 6).

Hepatic levels of PCNA, an index of cell proliferation and regeneration, were significantly (*p* < 0.05) decreased in the sepsis group (Figure 5). This finding aligns with the observed elevation in hepatic apoptosis in these animals (Figure 4). Menthol administration significantly (*p* < 0.05) attenuated the sepsis-induced decline in PCNA expression (Figure 5).

### 3.6 Analysis of correlation between different parameters

**FIGURE 6:**
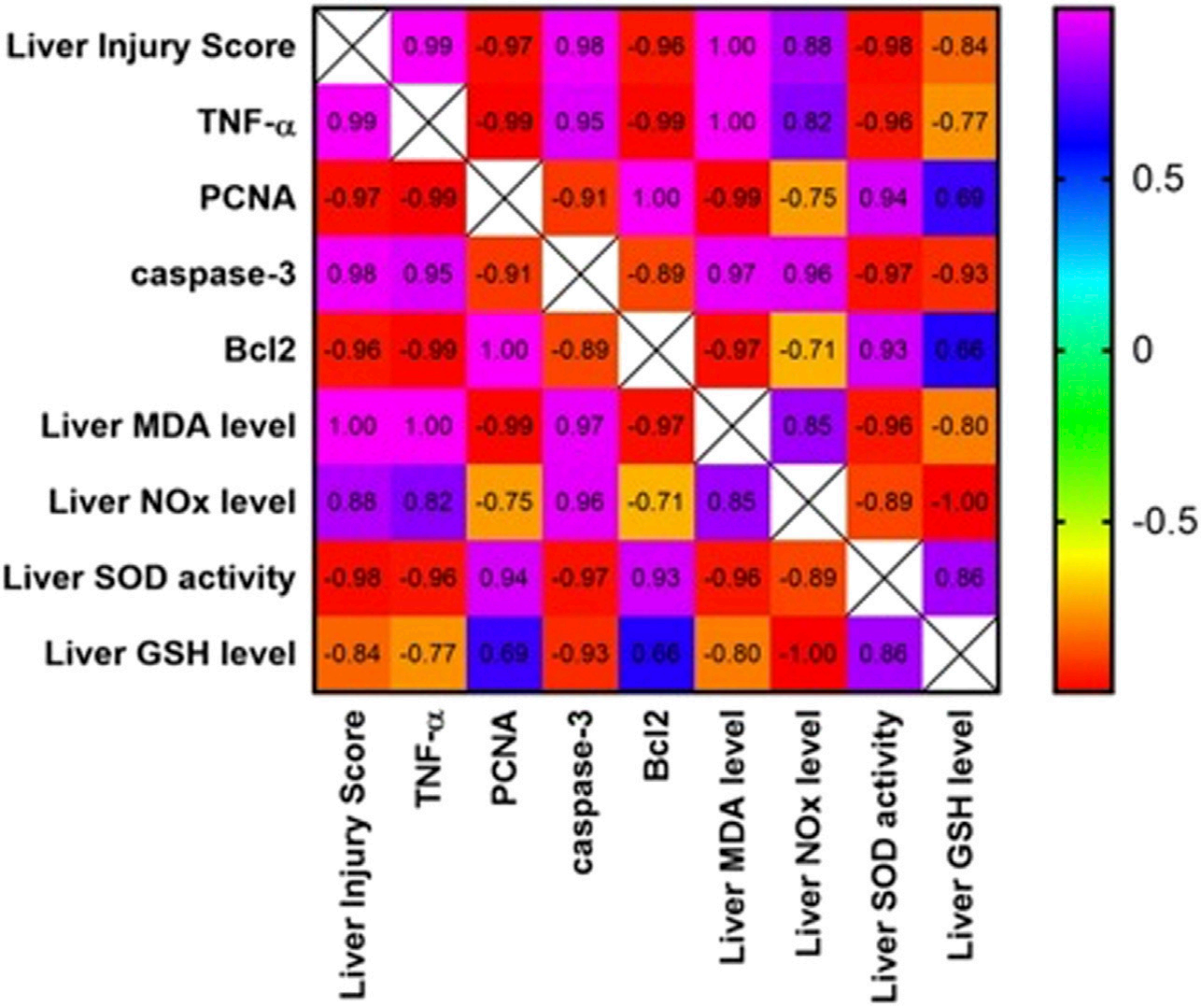
Correlation matrix for different parameters included in the study. Pearson correlation coefficient (r) was used to measure the correlation. If r is between |0.3| and |0.7|, a moderate correlation is indicated. If r >|0.7|, a strong correlation is indicated. If r<|0.3|, a weak correlation is indicated. Positive values indicate a positive correlation, while negative values indicate a negative correlation. The color of the scale bar to the right ranges from violet to red, indicating r values from +1 to -1. The graph is colored according to the scale. TNF-α: tumor necrosis factor-alpha; PCNA: proliferating cell nuclear antigen; Bcl-2: B-cell lymphoma 2.

A strong positive correlation was found between the liver injury score and the inflammatory marker, TNF-α., and the apoptotic marker, cleaved caspase-3 (Figure 6). In contrast, the liver injury score was negatively correlated with the hepatocellular regeneration marker, PCNA, and the anti-apoptotic marker, Bcl-2. Furthermore, hepatic TNF-α and cleaved caspase-3 were positively correlated with each other and negatively correlated with the liver expression of Bcl-2 and PCNA.

## 4 Discussion

Sepsis is a life-threatening condition resulting from an unregulated host response to an infection. Sepsis causes damage and failure of many organs, including the heart, kidney, and liver. Despite sepsis induced lung injury is highly common than sepsis-induced hepatic injury, the latter is associated with a higher mortality rate (Yan and Li, 2014; Strnad et al., 2017; Woznica et al., 2018). Sepsis-induced hepatic dysfunction directly contributes to the poor prognosis and increased risk of death in septic patients (Yan and Li, 2014; Strnad et al., 2017; Woznica et al., 2018). The pathophysiology of sepsis-induced hepatic failure is extremely complex. However, a systemic hyperinflammatory response associated with increased oxidative stress contributes to the hepatic dysfunction (Liu et al., 2017). Here, we investigated, for the first time, the hepatoprotective effects of menthol, a powerful antioxidant and natural anti-inflammatory agent, in the CLP-induced sepsis model. Menthol (100 mg/kg, I. G) administration after CLP surgery decreased hepatic oxidative stress, inflammation, and apoptosis and enhanced hepatocellular regeneration.

In our current study, which is consistent with previous studies (Ahmed et al., 2020; Al-Kadi et al., 2020), the induction of sepsis by CLP resulted in severe hepatic injury manifested as fatty changes, infiltration of inflammatory cells, apoptotic and necrotic hepatocyte death, as well as the dilation and congestion of the central vein. Damage to the liver tissues was confirmed by the elevation in serum levels of hepatic transaminases (ALT and AST). These cytoplasmic enzymes are typically localized in the cytoplasm of hepatocytes, with minimal presence in the serum; however, hepatic injury increases their serum levels. Therefore, they are commonly used as surrogates of hepatic function, indicating the magnitude of hepatocellular injury (McGill, 2016). Their levels were markedly elevated in the early phase of sepsis due to sepsis-induced hypotension and hepatic hypoperfusion (Fuhrmann et al., 2009; Fuhrmann et al., 2010; Dou et al., 2019b). Treatment of CLP rats with menthol 2 h after surgery prevented the elevation of serum hepatic transaminases, which was supported by the preserved histopathology in this group. Menthol abrogated the sepsis-induced dilation and congestion of the central hepatic veins and decreased the necrosis and fatty changes of hepatocytes, suggesting a possible hepatoprotective effect against the CLP sepsis model. Notably, the hepatoprotective effects of menthol were previously reported against paracetamol- and CCl_4_-induced hepatic injury (Janbaz and Gilani, 2002).

The increased oxidative stress significantly contributes to the hepatic dysfunction induced by sepsis (Moradi et al., 2021). There are many sources of ROS production during sepsis, including the exaggerated release of inflammatory mediators, neutrophil infiltration, and mitochondrial dysfunction (Zhang et al., 2018). In this study, the sepsis group showed increased hepatic MDA levels. Oxidation of polyunsaturated phospholipids of the cell membrane under increased oxidative stress results in the formation of MDA. Thus, it is used as a standard marker of increased oxidative stress (Petronilho et al., 2015; Zhou et al., 2020). The sepsis group also showed elevated total nitrite levels, indicating high tissue levels of the vasodilator mediator, nitric oxide (NO). Elevated NO mediates the sepsis-induced hypotension, and when combined with ROS, contributes to the formation of peroxynitrite, a potent oxidizing agent that causes cellular damage (Iwakiri and Kim, 2015). The current results align with the previous studies indicating that sepsis-induced ROS production parallels a decrease in hepatic antioxidant defense markers, such as GSH and SOD (Giustina et al., 2019; Larrouyet-Sarto et al., 2020). It is worth mentioning, that our study lacks the measurement of other important antioxidative stress parameters such catalase and glutathione peroxidase enzyme activities. However, results from our previous work (Ahmed et al., 2020; Ibrahim et al., 2020a; Al-Kadi et al., 2020; Al‐Kadi et al., 2021; Senousy et al., 2022), in line with others (Chen et al., 2012; Yang et al., 2018; Giustina et al., 2019; Ibrahim et al., 2020b; Larrouyet-Sarto et al., 2020; Aboyoussef et al., 2021), showed that either enzyme alone or GSH is a good surrogate of the antioxidant capacity of the tissue, while the levels of MDA directly reflect tissue oxidative damage. Akin to its established antioxidant effects (Rozza et al., 2014; Bastaki et al., 2018), menthol ameliorated the sepsis-induced elevation in hepatic ROS and total nitrite levels, while preserving the hepatic antioxidant GSH and SOD levels. These effects were positively correlated with the observed menthol-induced improvement in hepatic functions and histopathology. Previous studies discussing menthol inhibition of neutrophil infiltration (Rozza et al., 2014), and the subsequent attenuation of ROS production, may explain the observed antioxidant effects of menthol.

Evidence supports crosstalk between oxidative stress and the initiation and progression of inflammation (Aziz et al., 2013; Pandey et al., 2015). Our data, in line with previous reports (Vandewalle et al., 2019; Ahmed et al., 2020; Al-Kadi et al., 2020), showed elevated hepatic TNF-α in the untreated septic rats, which positively correlated with increased hepatic oxidative stress and decreased antioxidant capacity. TNF-α enhances the expression of inducible nitric oxide synthase (iNOS) leading to a massive release of NO (Ozaki et al., 2010), the stimulation of ROS production, the expression of inflammatory cytokines such as IL-6, the activation of neutrophil infiltration, and ultimately hepatic damage (Bozza et al., 2007; Vandewalle et al., 2019). Bacteremia and subsequent endotoxemia directly stimulate the release of inflammatory cytokines, including TNF-α, IL-1β, IL-6, IL-12, and IL-18 in sepsis (Traeger et al., 2010). Our results showed downregulated hepatic TNF-α and decreased oxidative stress in the menthol-treated septic rats, supporting the immunomodulatory and anti-inflammatory effects reported by others (Rozza et al., 2014; Zaia et al., 2016). Accumulating evidence shows that the activation of the transient receptor potential melanin-8 (TRPM8) mediates the menthol-induced cooling sensation and analgesic effects (Bautista et al., 2007; Ordas et al., 2019). Interestingly, the activation of TRPM8 reduced the release of TNF-α and increased the anti-inflammatory cytokine IL-10 (Khalil et al., 2016; Wang et al., 2017; Khalil et al., 2018; Ordas et al., 2019), which further supports the anti-inflammatory effects observed with menthol in this study. Unfortunately, the current results cannot determine whether the activation of TRPM8 mediates the hepatoprotective effects of menthol. Thus, it would be of interest to design future studies to further explore the role of TRPM8 and its potential significance in novel anti-sepsis modalities.

Sepsis induces hepatocellular apoptosis leading to organ damage and dysfunction (Jaeschke et al., 2000; Gao et al., 2018). As a result, attenuation of apoptosis would counteract the sepsis-induced hepatocellular damage (Yoon and Gores, 2002). Tissue infiltration by neutrophils and macrophages contributes to the proapoptotic signaling by elevating the levels of cytokines (e.g., TNF-α), NO, and ROS (Zhang et al., 2014). Cleaved caspase-3, a hallmark of apoptosis, is activated by several cell death signals to execute the apoptotic changes (Mazumder et al., 2008). Our results showed increased hepatic expression of cleaved caspase-3, positively correlated with sepsis-induced liver injury. To add to that, the liver of septic rats revealed low expression of the anti-apoptotic marker, Bcl-2. The increased hepatocellular apoptosis observed in septic rats is coherent with previous studies (Wesche-Soldato et al., 2007; Su et al., 2020). As reported by other studies (Rozza et al., 2014), menthol exhibited remarkable anti-apoptotic effects; it nearly eliminated the sepsis-induced elevation in hepatic cleaved caspase-3 and upregulated the anti-apoptotic marker, Bcl-2. The anti-apoptotic, anti-inflammatory, and antioxidant effects of menthol support the observed protection against sepsis-induced hepatotoxicity.

There is a correlation between hepatocellular regeneration and the levels of PCNA, a nuclear factor involved in cell proliferation and DNA replication (Hall et al., 1990; Moldovan et al., 2007). Increased PCNA levels indicate hepatocyte regeneration and recovery from hepatic damage (Li et al., 2016). In addition, PCNA protects against apoptotic cell death by binding to and inactivating procaspases (Witko-Sarsat et al., 2010). In sepsis, increased hepatic inflammation and apoptosis decrease the gene expressions of PCNA, as shown in this study and other studies (Abcejo et al., 2011). The decreased hepatic PCNA levels in septic rats correlated with liver injury, inflammation, and apoptosis. Interestingly, menthol upregulated the hepatic expression of PCNA in CLP septic rats. This effect was positively correlated to the menthol-induced enhancement of hepatic antioxidant activity and decreased apoptotic and inflammatory effects, suggesting a potential role in the enhancement of hepatocellular regeneration.

In conclusion, we investigated, for the first time, the hepatoprotective effects of menthol in an experimental CLP model of sepsis. The antioxidant, anti-inflammatory, and anti-apoptotic effects of menthol contributed to its hepatoprotection. In addition, menthol may induce the expression of PCNA, thus, promoting compensatory liver regeneration. Together, these effects suggest that menthol is a promising therapy that limits liver injury in septic patients. Indeed, the lack of *in vitro* studies that further explore the hepatoprotective effects of menthol in sepsis, the need to investigate the possible involvement of the menthol receptor (TRPM8) in preventing sepsis-associated complications and the elucidation of the main signaling pathways mediating the antioxidant effects of menthol are considered limitations of the present study that should be addressed in future studies.

## Data Availability

The raw data supporting the conclusions of this article will be made available by the authors, without undue reservation.
