# Supplementary material for: Protective effects of menthol against sepsis-induced hepatic injury: Role of mediators of hepatic inflammation, apoptosis, and regeneration
Source: Front Pharmacol. 2022 Aug 30;13:952337. doi: 10.3389/fphar.2022.952337 (PMC9476320; doi:10.3389/fphar.2022.952337)
Supplement: Supplementary file 1 [file DataSheet1.pdf]

Supplementary data

Supplementary Figure 1**:** Photographs showing inflammation and ischemia at the site of cecum ligation in all CLP rats.

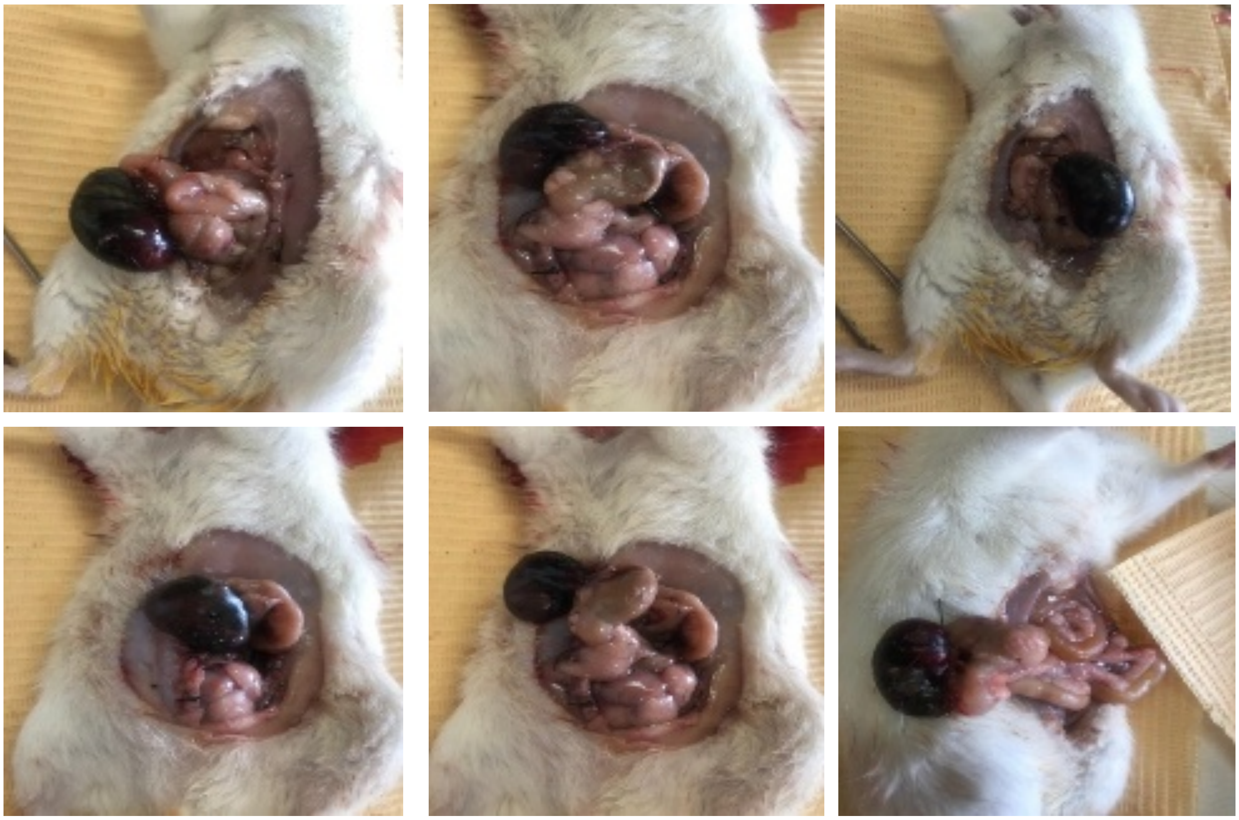

Supplementary Figure 2: Preliminary studies of survival of Menthol (50 mg/kg, I.G).

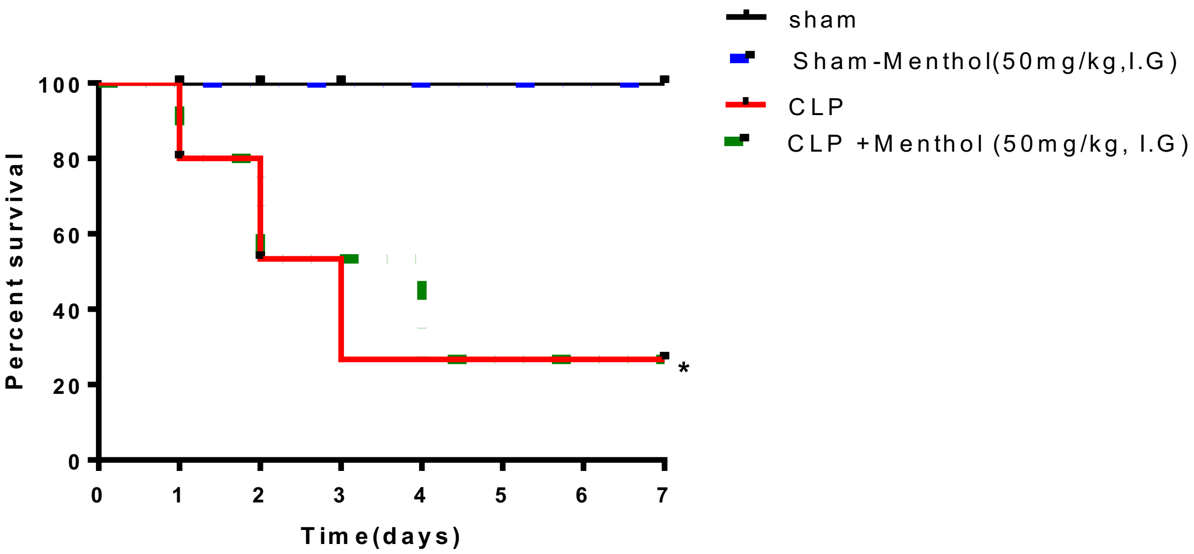

As shown in the supplementary Figure 1: administration of Menthol in a dose of (50 mg/kg, i.g) 2 h after performing CLP surgery resulted in (26.7%) by the end of the 7th day. CLP group showed (26.7%) survival at the end of the 7th day. * refers to a significant difference from Sham at ($p<0.05$).
